# Insights into 1-butyl-3-methylimidazolium hydrogen sulfate recovery from wastewater by electrodialysis with heterogeneous ion-exchange membranes

**DOI:** 10.1038/s41598-025-04108-y

**Published:** 2025-06-02

**Authors:** Dorota Babilas-Krzyżowska, Jitka Chromikova, Andrzej Milewski, Sandra Pluczyk-Małek, Piotr Dydo

**Affiliations:** 1 Department of Inorganic, Analytical Chemistry and Electrochemistry, Faculty of Chemistry, Silesian University of Technology, B. Krzywoustego 6 Street, 44-100 Gliwice, Poland; 2 Department of Environmental Engineering, Faculty of Mining and Geology, VŠB – Technical University of Ostrava, 17. Listopadu 15 Street, Ostrava-Poruba, Czech Republic; 3 Department of Physical Chemistry and Technology of Polymers, Faculty of Chemistry, Silesian University of Technology, M. Strzody 9 Street, 44-100 Gliwice, Poland; 4 Centre for Organic and Nanohybrid Electronics, Silesian University of Technology, S. Konarskiego 22B Street, 44-100 Gliwice, Poland

**Keywords:** Electrodialysis, Ionic liquids recovery, 1-Butyl-3-methylimidazolium hydrogen sulfate, Heterogeneous ion-exchange membranes, Chemical engineering, Environmental chemistry

## Abstract

Due to the negative impact of ionic liquids (ILs) on the environment and their high cost, it is very important to examine their recovery and recycling methods. Thus, in this study, research on 1-butyl-3-methylimidazolium hydrogen sulfate ([Bmim]HSO_4_) recovery from wastewater by electrodialysis (ED) is presented. The influence of the initial [Bmim]HSO_4_ concentration, the voltage, and the linear flow velocity on the effective [Bmim]HSO_4_ recovery was examined. To assess the effectiveness of the proposed method, factors such as IL percentage recovery, degree of concentration, electric current efficiency, and energy consumption were determined. It was found that as the initial concentration of [Bmim]HSO_4_ in the feed solution increased from 0.01 to 0.2 M, the [Bmim]HSO_4_ recovery efficiency increased. A 2.3-fold concentration of the [Bmim]HSO_4_ in the concentrate solution with a recovery of 98.8%, a current efficiency of 67.3%, and energy consumption of 28 kWh/m^3^ was achieved for ED of a 0.2 M [Bmim]HSO_4_ solution using heterogeneous ion-exchange membranes. Additionally, the voltage and linear flow velocity affected the [Bmim]HSO_4_ recovery. The highest ED efficiency for the recovery and concentration of [Bmim]HSO_4_ was obtained at an applied potential of 4 V, a 2 cm/s linear flow velocity, and 0.2 M IL in the feed solution.

**Supplementary Information:**

The online version contains supplementary material available at 10.1038/s41598-025-04108-y.

## Introduction

In recent years, the most popular class of ionic liquids (ILs) has been 1,3-dialkylimidazolium salts. One example of a 1,3-dialkylimidazolium salt with acidic hydrogens on the anion is 1-butyl-3-methylimidazolium hydrogen sulfate ([Bmim]HSO_4_). This compound is mainly produced by a chemical reaction between 1-butyl-3-methylimidazolium chloride and sulfuric acid, with removal of the volatile hydrochloric acid^1^. [Bmim]HSO_4_ is characterized by a density of 1.27 g/cm^3^ and a melting point of 28 °C, as well as a low toxicity, due to which it is commonly used in many industries^2,3^. [Bmim]HSO_4_ can be applied, for example, as a catalyst and solvent^4^. It is used as a catalyst in many reactions, such as a thioacetalization and an acetalization of carbonyl compounds and their subsequent deprotection^5^; a one-pot three-component synthesis of amidoalkyl naphthols with 96% yields at 60 °C and shorter reaction time compared to other tested ILs with the same imidazolium cation^6^; a transesterification of nannochloropsis to fatty acid methyl esters^7^; a one-pot three-component synthesis of 2,3-dihydro-4(1H)-quinazolinones with 85% yield^8^; and oleic acid esterification for green biodiesel synthesis^9^. According to the available literature, acidic ILs with acidic hydrogens on the anion can improve biomass conversion and fractionation^4,7^. [Bmim]HSO_4_ can, therefore, also be used as a catalyst in the conversion of bamboo shoot shell to levulinic acid^10^. [Bmim]HSO_4_ was successfully used as a catalyst in poplar and coir fractionation into cellulose materials. It was noted that [Bmim]HSO_4_ gave a delignification rate of 98%^11^. Moreover, [Bmim]HSO_4_, as an acidic IL, can be applied as an interface modulator for perovskite solar cells. It was found that [Bmim]HSO_4_ created a surface dipole and, in consequence, improved charge extraction and decreased the energy loss^12^. [Bmim]HSO_4_ is also applicable in the electroplating industry as an electrolyte additive^13–15^: in copper and zinc electrodeposition, the [Bmim]HSO_4_ additive allowed more leveled and fine-grained cathodic deposits to be obtained^13,14^.

Due to the wide applications of ILs in a variety of industrial fields, their high price, and a degree of toxicity, the development of ILs recovery methods is very important^16–20^. Moreover, European Union Directives recommend reusing and recovering such substances from wastewater^21^. The available ILs recovery methods can be classified based on their mechanism as phase addition methods, force field methods, or barrier methods^22^. An example of ILs recovery by phase addition is a salting-out process in which an electrolyte is added as a saturated solution or solid to wastewater^22,23^. Recovery using a force field can be achieved, for example, by a magnetic field or by gravity settling and centrifugation. Whereas membrane processes are examples of ILs recovery using barrier methods^22^.

In the existing literature, methods of [Bmim]HSO_4_ recovery from solutions include extraction, distillation, and integrated processes of precipitation, filtration, and evaporation^1–4^. For example, [Bmim]HSO_4_ applied in the pretreatment and fractionation of wheat straw could be recovered by the neutralization of the obtained process filtrate with sodium hydroxide. In the next step, the obtained aqueous solution with the IL and NaCl was evaporated, and the IL and NaCl were precipitated. The IL was then dissolved in acetonitrile, and in consequence NaCl was removed by filtration as an insoluble residue. The acetonitrile was then removed from the IL by evaporation, and the recovered [Bmim]HSO_4_ was then dried under vacuum^24^. [Bmim]HSO_4_ was also applied in biodiesel production, being recovered from the esterification step of the claimed method. In the first [Bmim]HSO_4_ recovery step, methanol was distilled off under vacuum. In second step, the mixture was cooled and centrifuged. In the third step, the [Bmim]HSO_4_ was separated after centrifugation and washed with ethyl acetate and hexane before being dried under vacuum^25^.

Unfortunately, these previously described methods consist of several stages and complicated operations. Moreover, the good miscibility of ILs with most solvents also reduces the application of conventional recovery methods. However, membrane processes can be very promising methods for ILs recovery, giving the advantage that there is no need to use additional reagents. In addition, membrane processes can be applied to the recovery of various types of ILs, unlike separation by magnetic force, which is limited to magnetic ILs^22,26,27^.

One membrane process that can be used for ILs recovery from aqueous solutions is electrodialysis (ED). ED is an electromembrane process capable of desalting the solution in the diluate compartment and, in consequence, concentrating the ionic salt in the concentrate compartment under the influence of an applied electrical potential difference. In this process, ions migrate from the diluate compartment to the concentrate compartment across ion-exchange membranes (IEMs) toward the appropriately polarized electrodes^28^. In the available literature, it was noted that due to the ionic nature of ILs, ED can be an efficient method for ILs recovery. ED has been successfully used for the recovery of imidazolium ILs such as [Amim]Cl, [Bmim]Cl, [Emim]Cl, and [Bmim]Br, as well as the non-imidazolium [TEA]HSO_4_^29–33^. It was concluded that the ILs recovery ratio could reach above 90% when ED was applied. Additionally, integrated membrane processes can be used for ILs recovery. Liang et al.^34^ used ultrafiltration and ED with bipolar membrane methods for [Bmim]HSO_4_ recovery from biomass pretreatment^34^. In the first step, the obtained [Bmim]HSO_4_ solution was diluted with water and purified from lignin and hemicellulose by ultrafiltration. In the next step, ED with a bipolar membrane was used for [Bmim]HSO_4_ regeneration^34^.

Due to the increasing potential of using acidic ILs in various industries and the promising application of membrane processes to ILs recovery, in this study, the basic research on the ED application for [Bmim]HSO_4_ recovery from aqueous solution were presented. The effect of the [Bmim]HSO_4_ concentration, voltage, and linear flow velocity on the effective [Bmim]HSO_4_ recovery were examined. Moreover, the IL recovery coefficient, the IL degree of concentration, the electric current efficiency (*CE*), and the energy consumption (*EC*) were determined to better understand the impact of process parameters and their reciprocal dependencies on the efficiency of [Bmim]HSO_4_ recovery. Also, the characteristics of ion-exchange membranes before and after ED and chemical stability of the recovered [Bmim]HSO_4_ were determined. Thus, discussed results could be an important milestone in planning further applied research on the acidic ILs recovery, and in development of insight for their recovery from actual industrial wastes. In addition, presented method could be of interest for the chemical engineering, biomass and wastewater treatment industries based on its potential for simultaneous recovery and concentration of [Bmim]HSO_4_ from industrial wastewater without causing any environmental harm, reduction of harmful and toxic effluent, and ease of operation.

## Materials and methods

### Reagents and analytical method

Experiments were conducted using diluate and concentrate aqueous solutions prepared by dissolving [Bmim]HSO_4_ (Sigma Aldrich, USA) in deionized water. The deionized water was prepared using a Millipore Elix 10 system, while 0.1 M H_2_SO_4_ was used as an electrode rinse solution.

The concentration of the [Bmim]HSO_4_ in experimental solutions were determined using a UV–VIS spectrophotometer (Shimadzu UV-2700i, Japan), the maximum absorption wavelength for the [Bmim]^+^ cation being 211.40 nm.

### ED setup

**Fig. 1:**
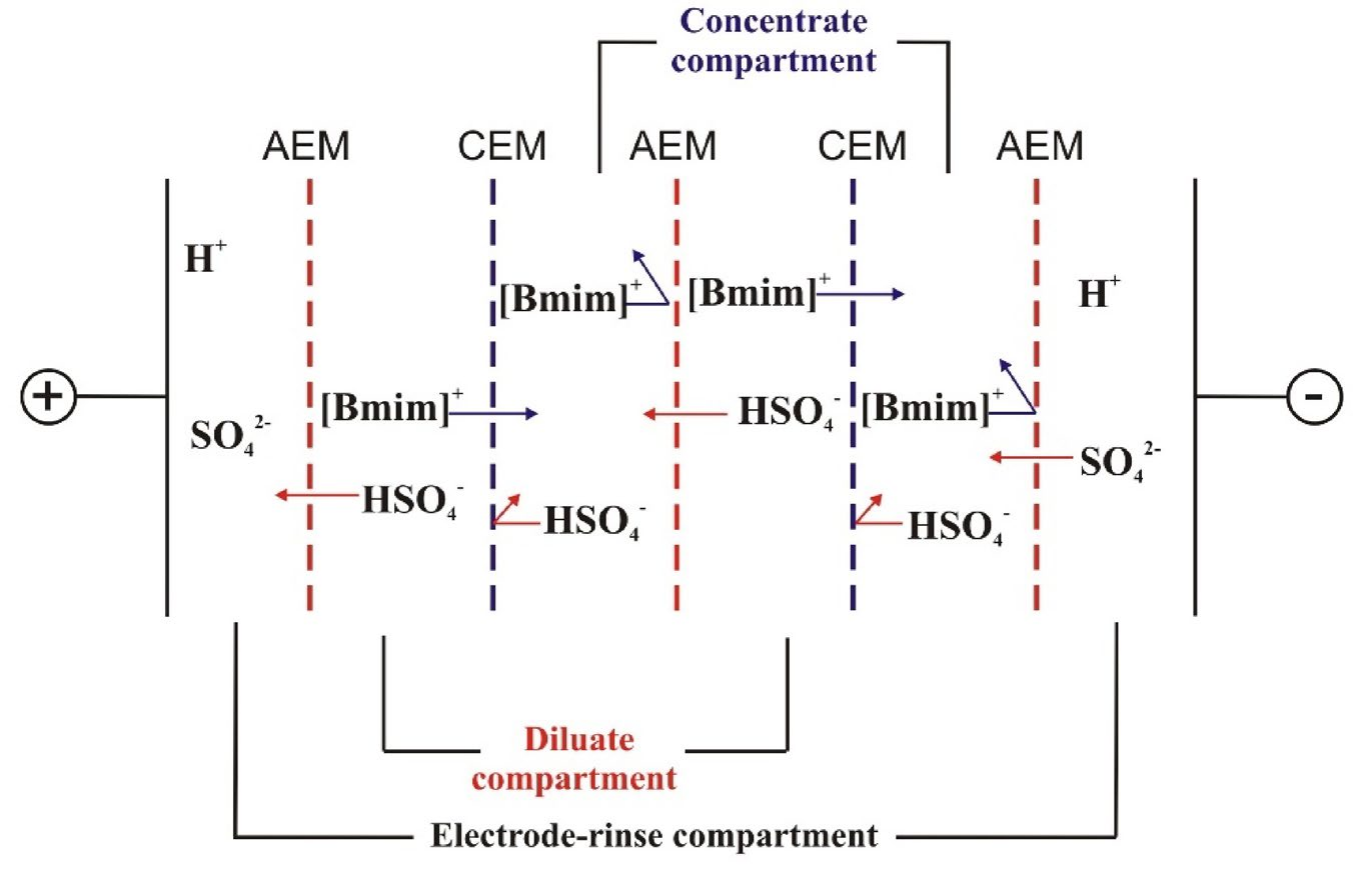
Detailed scheme of the ED stack for [Bmim]HSO_4_ recovery.

The experiments were carried out using a lab-scale ED module with an effective cation-exchange membranes area of 128 cm^2^. The effective area of a single membrane was 64 cm^2^. The ion-exchange membranes (IEMs) used in this study were heterogeneous AM(H)-CM(H) (Ralex, Czech Republic), while the anode and cathode in the ED module were made of platinized titanium. A detailed scheme of the ED module for [Bmim]HSO_4_ recovery is shown in Fig. 1.

All the experiments were conducted periodically with process solution recirculation**.** The process solutions were recirculated by a peristaltic pump (Masterflex L/S, Cole-Parmer, United States). The volumes of the experimental solutions were 200 mL for the diluate, 100 mL for the concentrate, and 200 mL for the electrode rinse solution. The experiments were performed until the diluate conductivity dropped below 1% of the initial diluate conductivity, which was monitored using a CPC-461 pH/conductivity meter (Elmetron, Poland).

Our research included a series of experiments on the effect of operating parameters, such as IL concentration (from 0.01 to 0.2 mol/L), applied voltage (from 2 to 4 V), and linear flow velocity (from 1 to 3 cm/s), on the ED performance.

### Membranes characteristics

The surface morphology of the tested membranes and their heterogeneity were investigated by using a scanning electron microscope (SEM, FEI Quanta 650 FEG, USA). SEM analysis was conducted with voltage settings at 20.00 kV. Morphological characterization of the membranes was also carried out using an atomic force microscopy (AFM, CoreAFM Nanosurf) with HQ:CSC17/Al BS contact mode AFM probe (MikroMasch). The surface root mean square roughness (*RMS*) and roughness average (*Ra*) were estimated using Gwyddion software, version 2.65, released 4 January 2024 (https://gwyddion.net/).

The hydrophilicity of the membranes was examined by an optical contact angle measuring device (OCA 15EC, DataPhysics, Germany). For this measurement, 1 μL of deionized water was dropped on the investigated surfaces, and the contact angle was measured by contour analysis. Three measurements from three independent samples were collected and averaged.

The FTIR spectra of the membrane samples before and after ED were recorded at room temperature using a Thermo Scientific™ Nicolet™ iS™5 FT-IR Spectrometer with Spectrum software version 6.3.2, equipped with a Nicolet 6700 Thermo Scientific™ iD7 ATR (attenuated total reflectance) sampling device containing a diamond/ZnSe crystal. The spectra were acquired from 4000 to 500 cm^−1^ at a scan speed of 0.2 cm/s with 8 × 16 (128 scans) an a resolution of 4 cm^−1^. A background spectrum was scanned under the same instrumental conditions before each series of measurements.

### ED data calculations

The performance and effectiveness of electrodialytic [Bmim]HSO_4_ recovery were assessed on the basis of the [Bmim]HSO_4_ recovery ratio ($R_{IL}$), the [Bmim]HSO_4_ degree of concentration (*R*_conc_), the electric current efficiency ($CE_{IL}$), as well as the energy consumption (*EC*) as follows:

(a) [Bmim]HSO_4_ recovery ratio

1 $$R_{IL} = \frac{{m_{IL,t}^{conc} }}{{m_{IL,0}^{dil} }} \cdot 100\%$$

where $m_{IL,t}^{conc}$ is the increase in the [Bmim]HSO_4_ mass [g] in the concentrate solution after ED, and $m_{IL,0}^{dil}$ is the initial mass [g] of the [Bmim]HSO_4_ in the diluate solution before ED.

(b) [Bmim]HSO_4_ degree of concentration

2 $$R_{conc} = \frac{{C_{IL,t}^{{conc_{{}} }} }}{{C_{IL,0}^{{dil_{{}} }} }} \cdot 100\%$$

where $C_{IL,t}^{{conc_{{}} }}$ is the final concentration [mol/L] of the [Bmim]HSO_4_ in the concentrate solution after ED, and $C_{IL,0}^{dil}$ is the initial concentration [mol/L] of the [Bmim]HSO_4_ in the diluate solution before ED.

(c) The electric current efficiency ($CE_{IL}$)

3 $$CE_{IL} = \frac{{F \cdot z \cdot \frac{{C_{IL,t}^{{conc_{{}} }} }}{{M_{IL} }} \cdot V_{conc,t} }}{{n \cdot \int\limits_{0}^{t} {I(t)dt} }} \cdot 100\%$$

where *F* is the Faraday constant (96,485 C·mol^−1^), *z* is the charge number of [Bmim]^+^, $V_{conc,t}^{{}}$ is the volume of the concentrate solution after ED [L], $C_{IL,t}^{{conc_{{}} }}$ is the increase in concentration of the [Bmim]^+^ in the concentrate solution after ED [g L^−1^], $M_{IL}$ is the molar mass of [Bmim]^+^ [g mol^−1^], *n* is the number of membrane pairs, and *I* is the electric current [A].

(d) The energy consumption (EC)

4 $$EC = \frac{{U \cdot \int\limits_{0}^{t} {I(t)dt} }}{{V_{dil,0} }}$$

where *EC* is the energy consumption, *U* is the applied voltage [V], *I* is the electric current in [A], and $V_{dil,0}^{{}}$ is the initial diluate volume [m^3^].

### Recovered [Bmim]HSO_4_ chemical stability

The chemical stability of the recovered [Bmim]HSO_4_ was determined by Nuclear Magnetic Resonance (NMR). ^1^H NMR spectra were recorded in D_2_O on a Varian 400 spectrometer, operating at 400 MHz, in which 3-(trimethylsilyl)propionic-2,2,3,3-d4 acid (TMSP-d4, sodium salt > 98%, Acros Organics) was used as the calibration standard. In this study, D_2_O was directly added to samples of the process solutions at various volumes to obtain 0.100 mmol/L of [Bmim]HSO_4_.

## Results and discussion

ED is a highly attractive technology for the recovery of ionic species from wastewater streams. The aim of this study is to evaluate the possibility of [Bmim]HSO_4_ recovery by ED method. It is known that the performance of ED depends on its essential operating parameters. Thus, the effect of IL concentration in the diluate solution, the applied voltage, and the linear flow velocity on the ED performance and [Bmim]HSO_4_ recovery are discussed below. Furthermore, the chemical stability of [Bmim]HSO_4_ in ED with heterogeneous IEMs is evaluated.

### Effect of IL concentration

The experiments on the effect of the initial [Bmim]HSO_4_ concentration included a series of five electrodialyses with different initial [Bmim]HSO_4_ concentrations in the diluate (0.01, 0.05, 0.1, 0.15, and 0.2 mol/L), at a linear flow velocity of 2 cm/s, and constant voltage potential of 4 V. The detailed experimental conditions are shown in Table 1.

**Table 1:** The initial diluate and concentrate chemical composition and experimental conditions.

Exp. No.	Initial diluate	Initial concentrate	Applied voltage, V	Linear flow velocity, cm/s
1	200 mL of 0.01 M [Bmim]HSO_4_	100 mL of 0.01 M [Bmim]HSO_4_	4	2
2	200 mL of 0.05 M [Bmim]HSO_4_	100 mL of 0.05 M [Bmim]HSO_4_	4	2
3	200 mL of 0.1 M [Bmim]HSO_4_	100 mL of 0.1 M [Bmim]HSO_4_	4	2
4	200 mL of 0.15 M [Bmim]HSO_4_	100 mL of 0.15 M [Bmim]HSO_4_	4	2
5	200 mL of 0.2 M [Bmim]HSO_4_	100 mL of 0.2 M [Bmim]HSO_4_	4	2

**Fig. 2:**
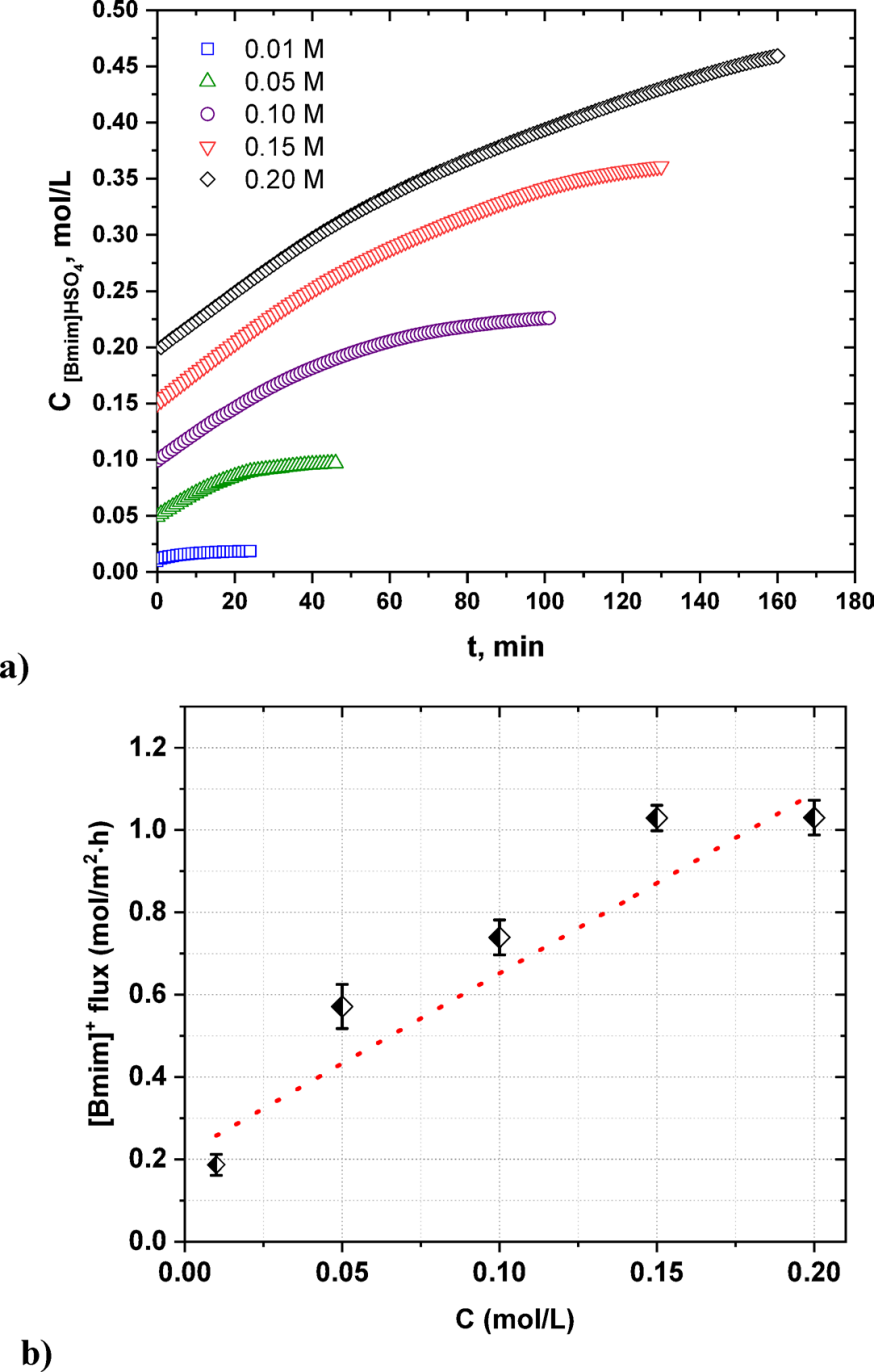
Influence of the feed concentration on the [Bmim]HSO_4_ distribution in the concentrate solution (**a**), and [Bmim]^+^ molar flux (**b**).

**Fig. 3:**
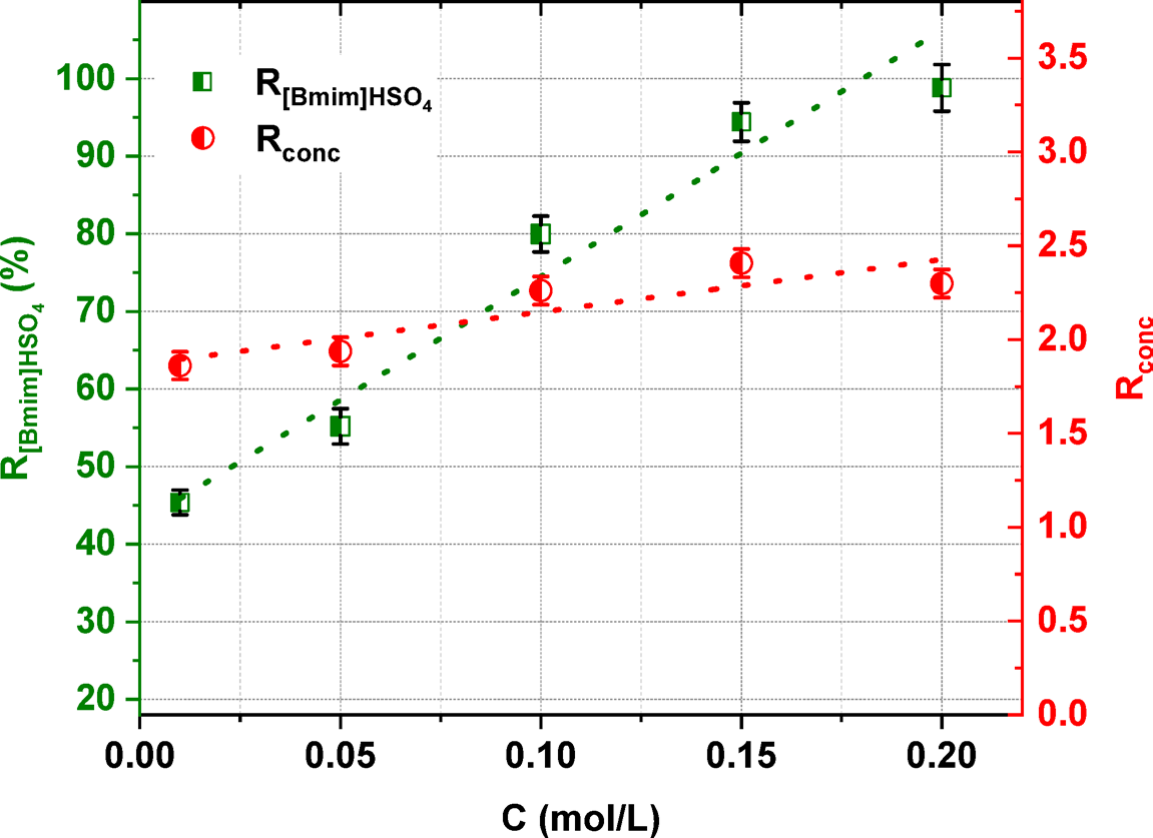
The influence of the [Bmim]HSO_4_ concentration in the feed solution on the IL recovery and its degree of concentration.

ED allows the transport of salts across IEMs and their concentration in the concentrate compartments^35^. Thus, as shown in Fig. 1, during the ED of [Bmim]HSO_4_ solution, the [Bmim]^+^ cations migrated from the diluate through the cation-exchange membranes to the concentrate compartment, while the HSO_4_^−^ anions migrated through the anion-exchange membranes to the concentrate compartments, so the content of [Bmim]HSO_4_ in the concentrate compartment increased (Fig. 2a). In Fig. 2 the influence of the feed concentration on the [Bmim]HSO_4_ distribution in the concentrate during the ED is presented. Generally, it was found that the quantity of [Bmim]HSO_4_ transported across the IEMs to the concentrate chamber and the desalination rate increased with an increase in the IL concentration in the feed solution, showing an increased mobility of [Bmim]HSO_4_ across IEMs for solutions with a high ionic strength. In Fig. 2a it can be clearly seen that the [Bmim]HSO_4_ concentration in the concentrate compartments during ED increased with an increase in the [Bmim]HSO_4_ concentration in the initial diluate. However, by the end of the ED, the increase in the [Bmim]HSO_4_ concentration in the concentrate was slower and no longer linear. As was suspected, the initial feed concentration influenced the desalination time, which increased with an increase in the feed concentration. At higher initial diluate concentrations, a higher mass had to be transferred across the IEMs. Moreover, this correlation could also be explained by the large difference in the electrical resistance of the solutions between the diluate and concentrate chambers at the end of ED. It was also noted that the [Bmim]^+^ flux and the [Bmim]HSO_4_ recovery ratio increased with increasing concentration of [Bmim]HSO_4_ (see Figs. 2b, 3). It was found that when the initial [Bmim]HSO_4_ concentration increased from 0.01 M to 0.2 M, the [Bmim]^+^ flux increased from 0.19 to 1.03 mol/m^2^∙h.

The influence of the [Bmim]HSO_4_ concentration in the feed solution on the IL recovery and the degree of concentration is shown in Fig. 3. It was noted that the concentration of the [Bmim]HSO_4_ in the feed solution had an effect on the recovery and the degree of concentration. In Fig. 3, it can be clearly seen that the [Bmim]HSO_4_ recovery increased with an increasing initial concentration of the [Bmim]HSO_4_ in the diluate. The [Bmim]HSO_4_ recovery rate increased from 45.4 to 98.8% when the initial [Bmim]HSO_4_ concentration in the feed solution increased from 0.01 to 0.2 mol/L. The highest recovery ratio was obtained for ED of the feed solution with a [Bmim]HSO_4_ concentration equal to 0.2 mol/L (in the examined range). The lowest [Bmim]HSO_4_ recovery was observed in the examined range for ED of a feed solution with a [Bmim]HSO_4_ concentration of 0.01 mol/L. This result could be explained by the incorporation of [Bmim]HSO_4_ in the IEMs during ED. The obtained results also shown that the [Bmim]HSO_4_ degree of concentration slightly increased with an increase in the initial diluate concentration. The degree of concentration reached 2.41 for 0.15 M [Bmim]HSO_4_ (Fig. 3).

**Fig. 4:**
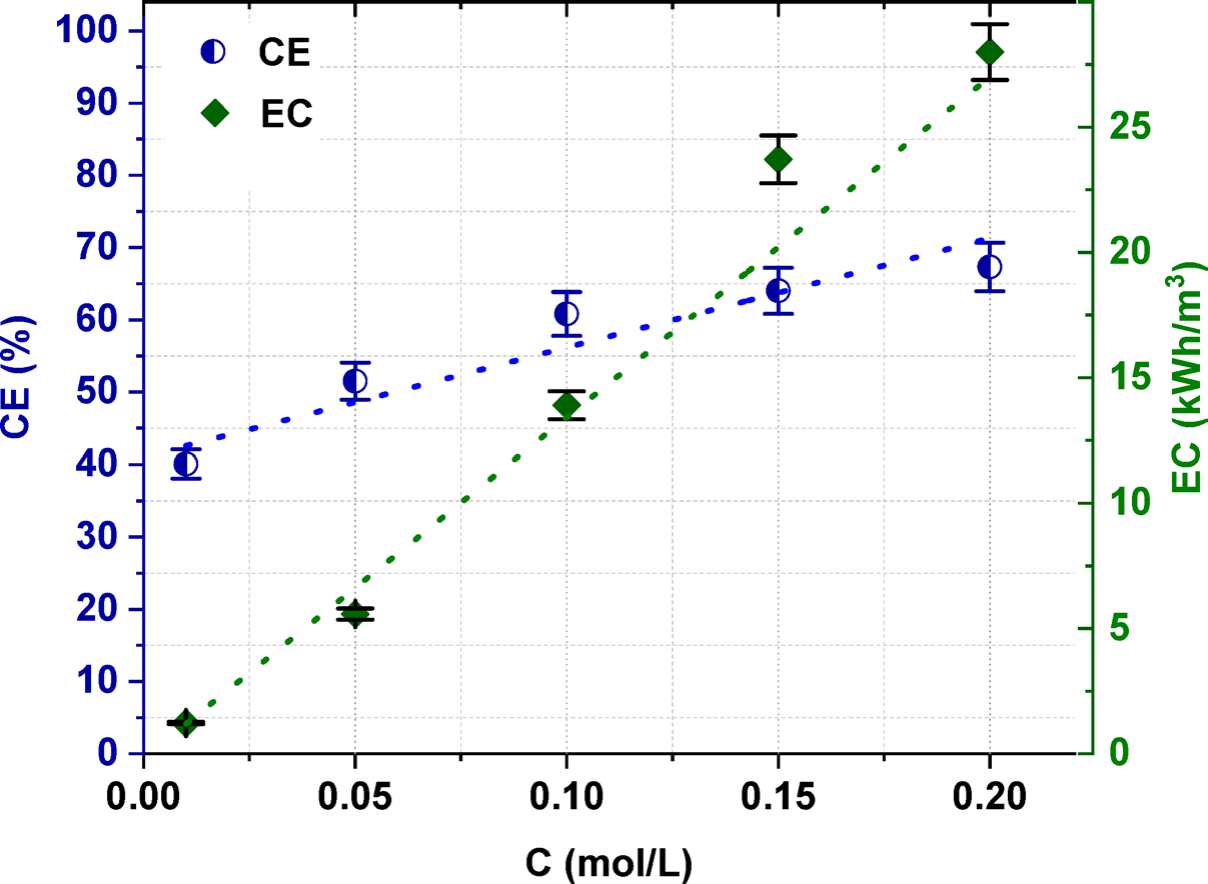
The influence of the [Bmim]HSO_4_ concentration in the feed solution on the electric current efficiency and energy consumption.

The initial concentration of the [Bmim]HSO_4_ in the diluate solution also had an effect on the electric current efficiency (*CE*) and energy consumption (*EC*) as shown in Fig. 4. As the concentration of [Bmim]HSO_4_ in the initial diluate increased, the CE increased. The CE could reach 67.3% with a feed concentration of 0.2 mol/L, a linear flow velocity of 2 cm/s, and a constant potential of 4 V. This result could be explained by the chemical character of the [Bmim]HSO_4_, which is an acidic IL. The electric current was also used for hydrogen cation transport across the IEMs. The concentration of ionic liquid correlates with the current efficiency calculated based on [Bmim]^+^ ions transport. However, part of the current is always carried by hydrogen ions migrating through the IEMs. This part would be relatively constant; for example, the same diffusional leak of hydrogen ions across outer anion-exchange membrane could be expect. At high concentration of [Bmim]^+^ ions, most the electric current was carried by [Bmim]^+^ flow, resulting in higher current efficiency. Moreover, the higher molecular weight of [Bmim]^+^ cation in comparison to simple ions may cause their slower transport across IEMs in the electric field. [Bmim]^+^ has higher ionic radius than simple cations such as Na^+^^36,37^. As shown in Fig. 4, the initial concentration of IL in the feed solution had some effect on the ED energy consumption. The energy needed to treat 1 m^3^ of feed solution increased with an increasing [Bmim]HSO_4_ content in the initial diluate. The *EC* for electrodialytic [Bmim]HSO_4_ recovery was shown to vary between 1.23 kWh/m^3^ for ED of 0.01 M feed solution to 28 kWh/m^3^ for ED of 0.2 M feed solution. This result can be explained by the longer time of desalination and the back diffusion effect when the feed concentration increased.

The obtained results are in agreement with refs.^30,38^. Table 2 shows a comparison of [Bmim]Cl and [Bmim]HSO_4_ recovery by ED. The results presented in Table 2 confirm that the concentration of IL in the initial diluate influenced the effectiveness of IL recovery from wastewater, the observed [Bmim]Cl recovery ratio increasing as the initial IL content in the diluate increased. Trinh et al.^30^ noted that the [Bmim]Cl recovery ratio increased from 37.7 to 70.7% when the initial diluate concentration increased from 0.013 to 0.04 M. Moreover, Bai et al.^38^ also investigated the effect of the initial [Bmim]Cl content in the diluate on the ED efficiency, but for a higher IL concentration from 0.2 to 0.6 M. It was proven that the IL recovery could reach 98% when the initial IL content in the diluate exceeded 0.2 M. The [Bmim]HSO_4_ recovery ratios in the present study agree well with the discussed imidazolium chloride IL recovery ratios. Other ED efficiency parameters, such as transport rate and *CE*, were also highly dependent on the IL content in the initial diluate. The results presented in Table 2 confirmed that the transport rate and *CE* increased with increasing imidazolium IL concentration in the initial diluate.

**Table 2:** Comparison of the [Bmim]Cl and [Bmim]HSO_4_ recovery by ED method.

IL name	IL concentration in feed solution	Voltage, V	Diluate to concentrate volume ratio	ILs recovery, %	Transport rate, mol/h·m^2^	Current efficiency, %	Ref.
[Bmim]Cl	0.013 M	10	1:1	37.7	0.23	57.4	^30^
	0.025 M	10	1:1	53.3	0.45	63.5	
	0.040 M	10	1:1	70.7	0.46	74.1	
[Bmim]Cl	0.2 M	25	1:1	98.5	1.05	95	^38^
	0.3 M	25	1:1	98.8	0.95	94	
	0.6 M	25	1:1	99.4	1.00	83	
[Bmim]HSO_4_	0.01 M	4	2:1	45.4	0.19	40.1	This study
	0.05 M	4	2:1	55.2	0.57	51.5	
	0.1 M	4	2:1	79.9	0.74	60.8	
	0.15 M	4	2:1	94.4	1.02	64	
	0.2 M	4	2:1	98.8	1.03	67.3	

Table 2 shows that the transport rate increased from 0.23 to 1.05 mol/h m^2^ when the initial concentration of [Bmim]Cl in the diluate increased from 0.013 M to 0.2 M^30,38^. This results were also in a good agreement with the [Bmim]HSO_4_ transport rate across IEMs. The [Bmim]HSO_4_ transport rate across the IEMs increased from 0.19 to 1.03 mol/h m^2^ when the initial concentration of [Bmim]Cl in the diluate increased from 0.01 to 0.2 M. This behavior can be explained by the presence of the same IL cation, which migrated through the cation-exchange membranes. The *CE* of [Bmim]Cl desalination, similarly to the desalination of [Bmim]HSO_4_, increased as the IL content in initial diluate increased. However, due to the higher pH of [Bmim]Cl solution than [Bmim]HSO_4_, the electrodialytic *CE* of [Bmim]Cl desalination was higher.

### The effect of applied voltage

**Fig. 5:**
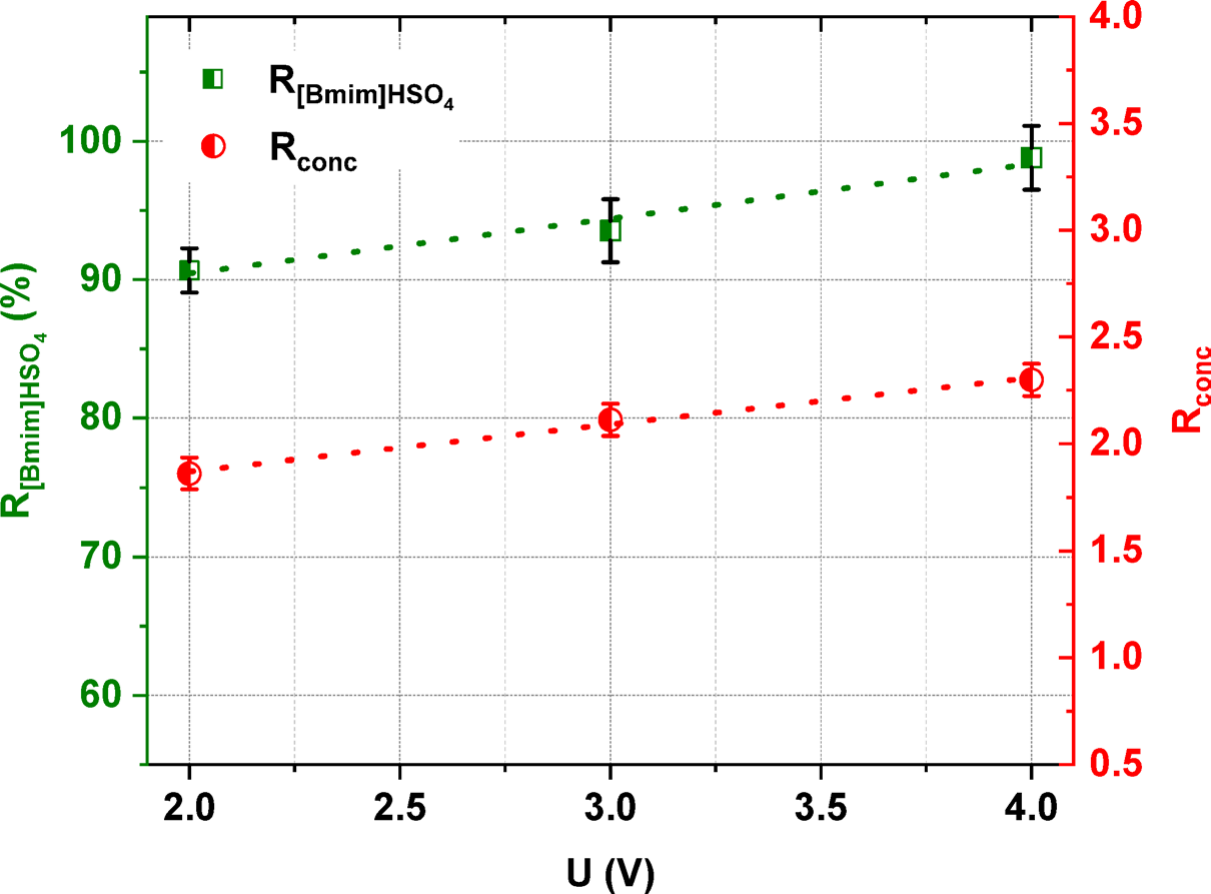
The influence of the applied voltage on the [Bmim]HSO_4_ recovery and its degree of concentration.

The electrical potential difference is a driving force of ED. Thus, the applied voltage being one of the crucial parameters in the ED method^39^. In this study, the effect of applied voltage was determined at 2, 3, and 4 V. The investigations were conducted with an initial [Bmim]HSO_4_ concentration of 0.2 mol/L and a linear flow velocity of 2 cm/s. Figure 5 illustrates the influence of applied voltage on the [Bmim]HSO_4_ recovery and its degree of concentration. It was noted that the [Bmim]HSO_4_ recovery increased as the applied voltage increased. This observation highlights the effect of voltage on the effective IL recovery by ED method. According to the Nernst-Planck equation, voltage is a driving force for the ion migration across IEMs^39^. Higher voltage leads to faster ion migration and desalination of the feed solution. It was shown in Fig. 5 that the [Bmim]HSO_4_ recovery reached 98.8% at 4 V, which was significantly higher compared with 90.7% at 2 V. Thus, the optimum voltage value in the examined range was 4 V. In Fig. 5, it can also be clearly seen that the applied voltage had some effect on the concentration degree of [Bmim]HSO_4_, which slightly increased with increasing voltage and in consequence with increasing electric current density. Due to the increasing electric current density, the ions were transported faster across IEMs^40–42^. Thus, increasing the cell pair voltage slightly increased the degree of concentration of the IL from 1.86 to 2.3. In addition, the high current density allowed the resistance in the membrane and the boundary layer to be limited.

**Fig. 6:**
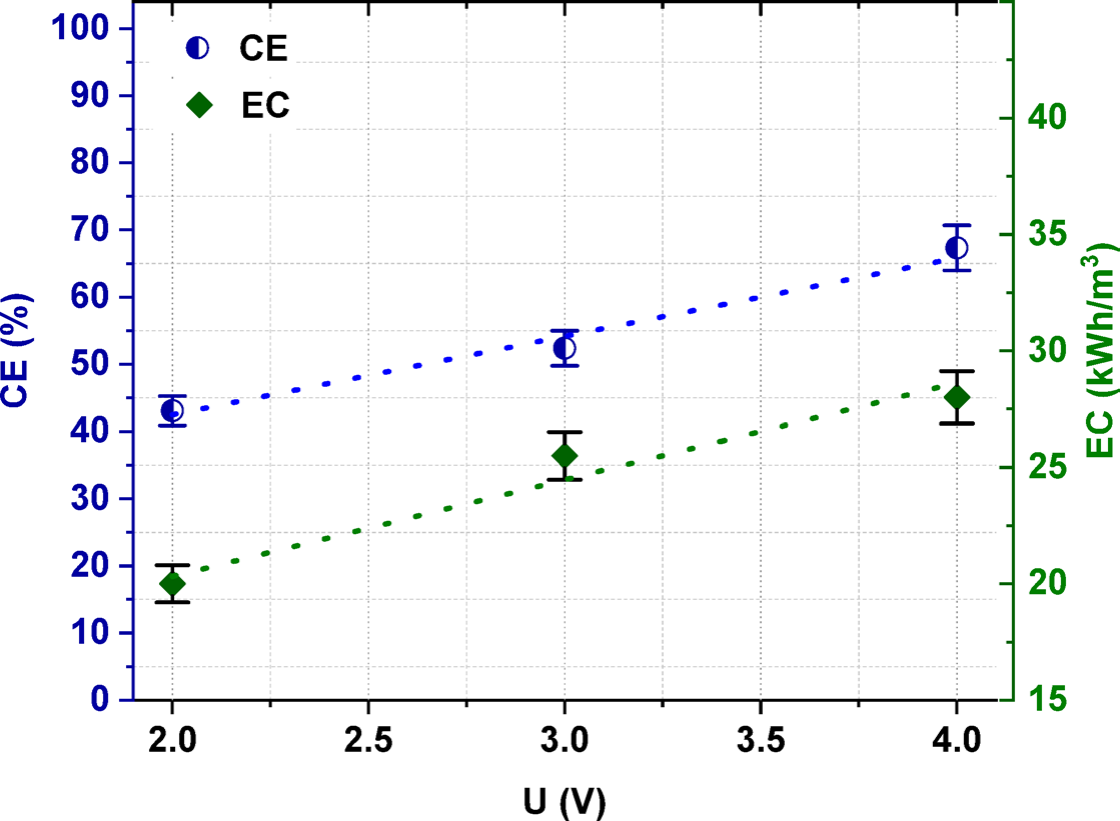
The influence of the applied voltage on the electric current efficiency and energy consumption.

As shown in Fig. 6, the applied voltage had some effect on the *CE* and *EC*. With an increase in the applied voltage, the *CE* increased from 43.1% to 67.3%. Also, the *EC* increased when the applied voltage increased. However, ED at lower voltages led to lower values for [Bmim]HSO_4_ recovery.

### The effect of linear flow velocity

**Fig. 7:**
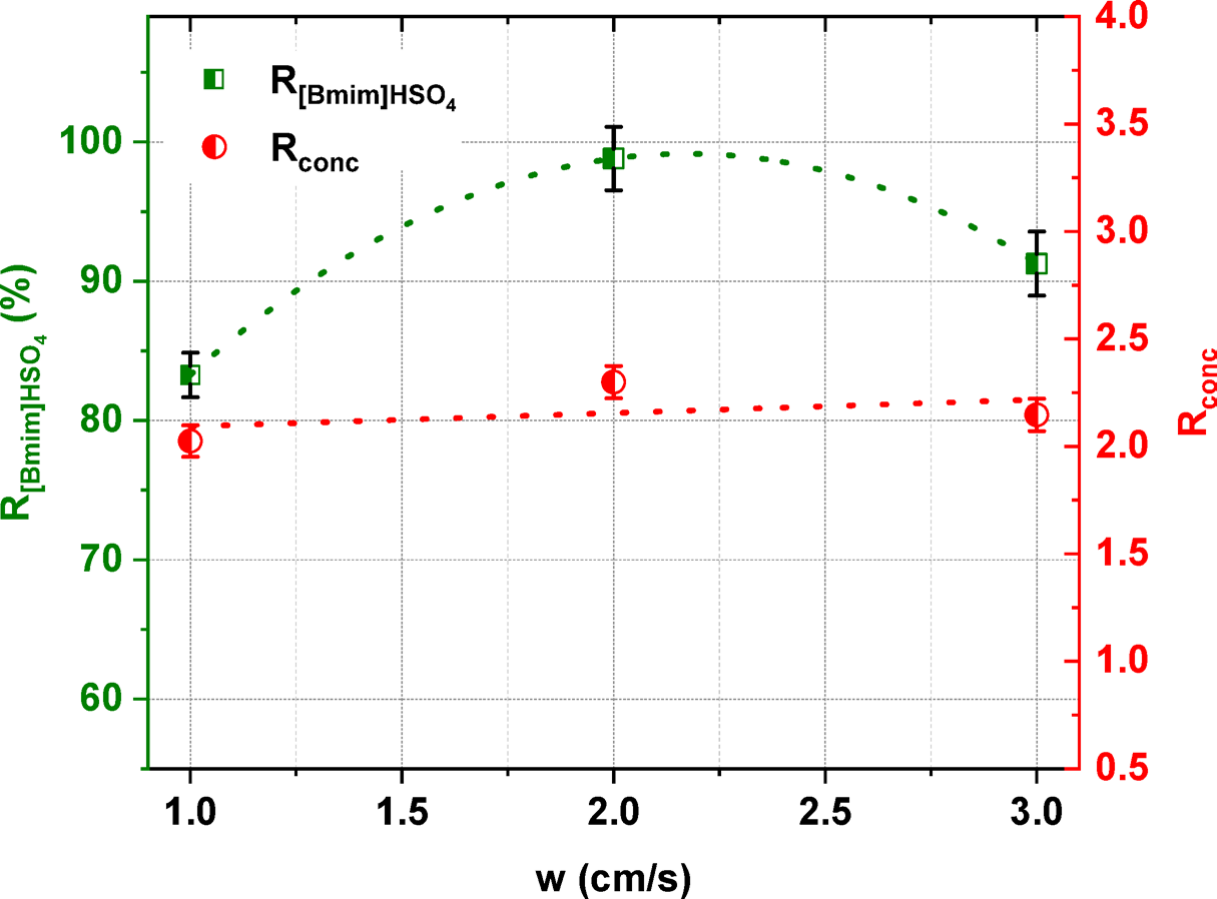
The influence of the linear flow velocity on the [Bmim]HSO_4_ recovery and its degree of concentration.

The linear flow velocity of diluate solution is a parameter that also plays an important role in the ED of [Bmim]HSO_4_. In general, the linear flow velocity has an effect on the mass transfer across IEMs and the thickness of the diffusion boundary layer^39,42^. The influence of the linear flow velocity on the [Bmim]HSO_4_ recovery and its degree of concentration is presented in Fig. 7. It was found that the [Bmim]HSO_4_ recovery increased to a maximum value of 98.8% as the linear flow velocity increased from 1 to 2 cm/s and then decreased when the linear flow velocity was 3 cm/s. This trend can be explained by the residence time of the solution in the compartments. When the linear flow rate increased, the residence time of the ions decreased. However, the thickness of the boundary layer adjacent to the membrane is known to decrease with the increasing linear flow velocity of a solution^43,44^. In Fig. 7, it was also found that the linear flow velocity did not significantly affect the degree of concentration of [Bmim]HSO_4_. It can be concluded that at higher linear flow velocities, the [Bmim]HSO_4_ recovery and degree of concentration decrease. As was mentioned before, the linear flow velocity refers to the residence time of ions in compartments and, in consequence their transport across IEMs. A higher flow rate means a reduced residence time and not enough time to transport ions across the IEMs^45^.

**Fig. 8:**
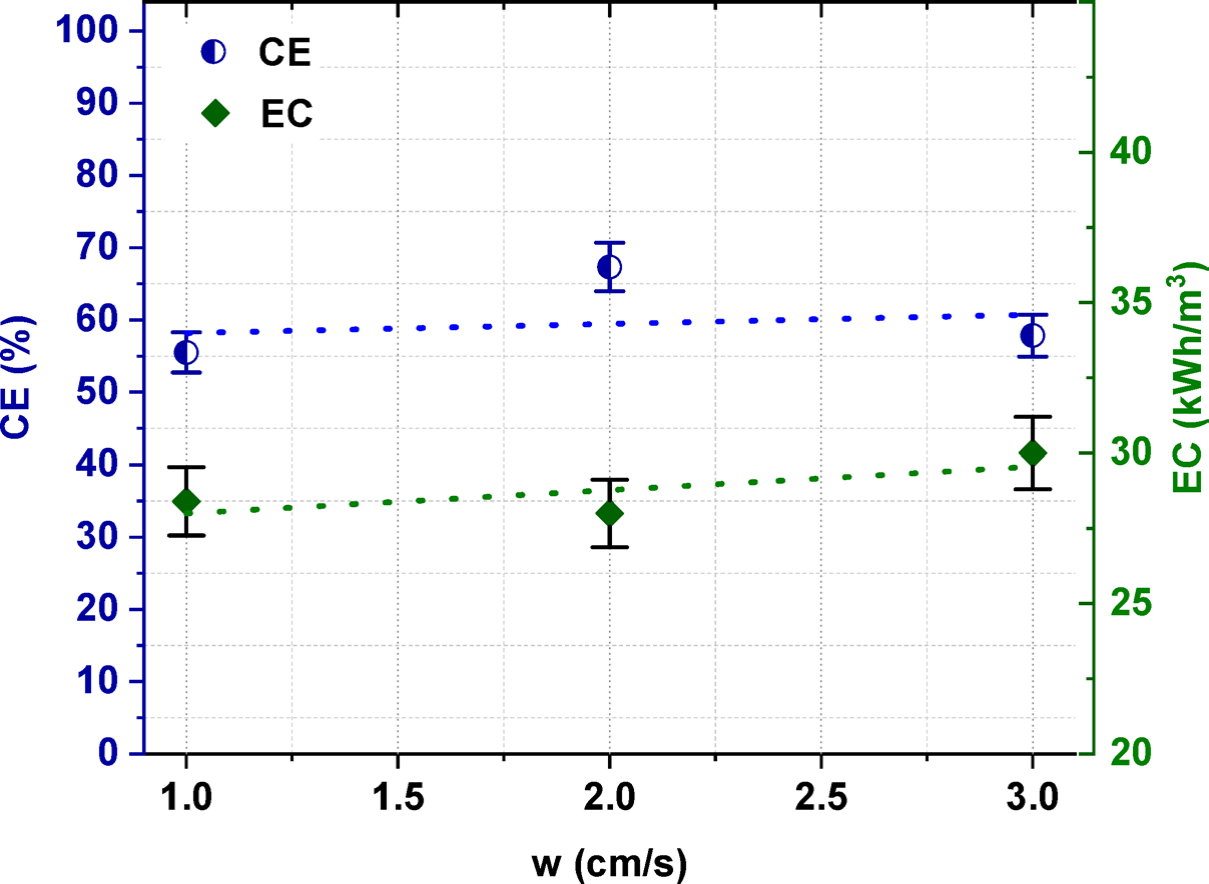
The influence of the linear flow velocity on the electric current efficiency and energy consumption.

The linear flow velocity influenced the *CE* and *EC*. When the linear flow velocity of the solutions increased from 1 to 2 cm/s, the *CE* increased from 55.5% to a maximum value of 67.3%, and then decreased to 57.8% when the linear flow velocity was 3 cm/s. The *CE* decreased at higher linear flow velocities because when the linear flow velocity increased, a lower residence time of ions in the compartment was observed, leading to a decrease in the total ions transport across the IEMs^43^. The *EC* also depended on the linear flow velocity of the diluate. As shown in Fig. 8, the *EC* increased with an increase in linear flow velocity, values in the range of 28.4 to 30 kWh/m^3^ being found. These results are in agreement with other studies^40,46,47^, where it was noted that increasing the linear flow velocity directly increases the electric current density and, in consequence, the *EC*.

Based on the obtained results, the optimal linear flow velocity was determined as 2 cm/s. ED at this linear flow velocity gave high [Bmim]HSO_4_ recovery with the highest degree of concentration and the lowest *EC* in the examined range.

### The characteristics of IEMs before and after ED

**Fig. 9:**
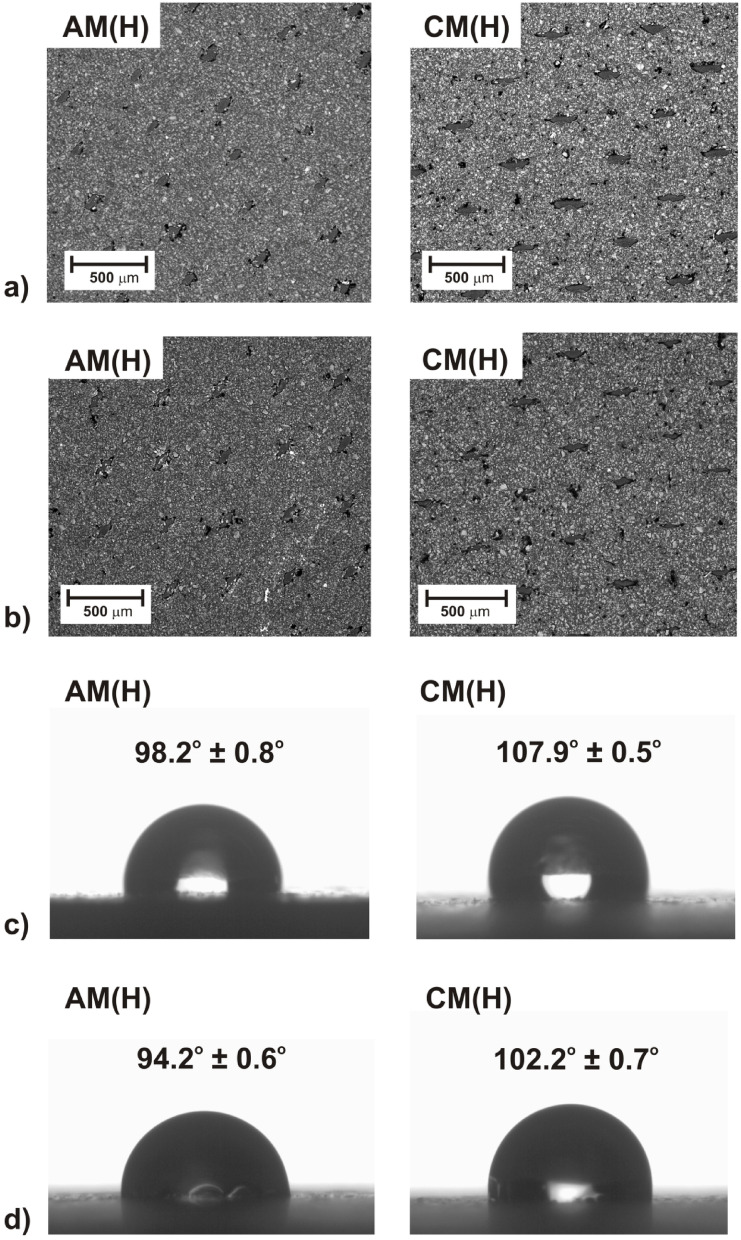
The surface morphology of the tested IEMs before (**a**) and after ED (**b**), and wettability of the membranes surface before (**c**) and after ED (**d**).

**Fig. 10:**
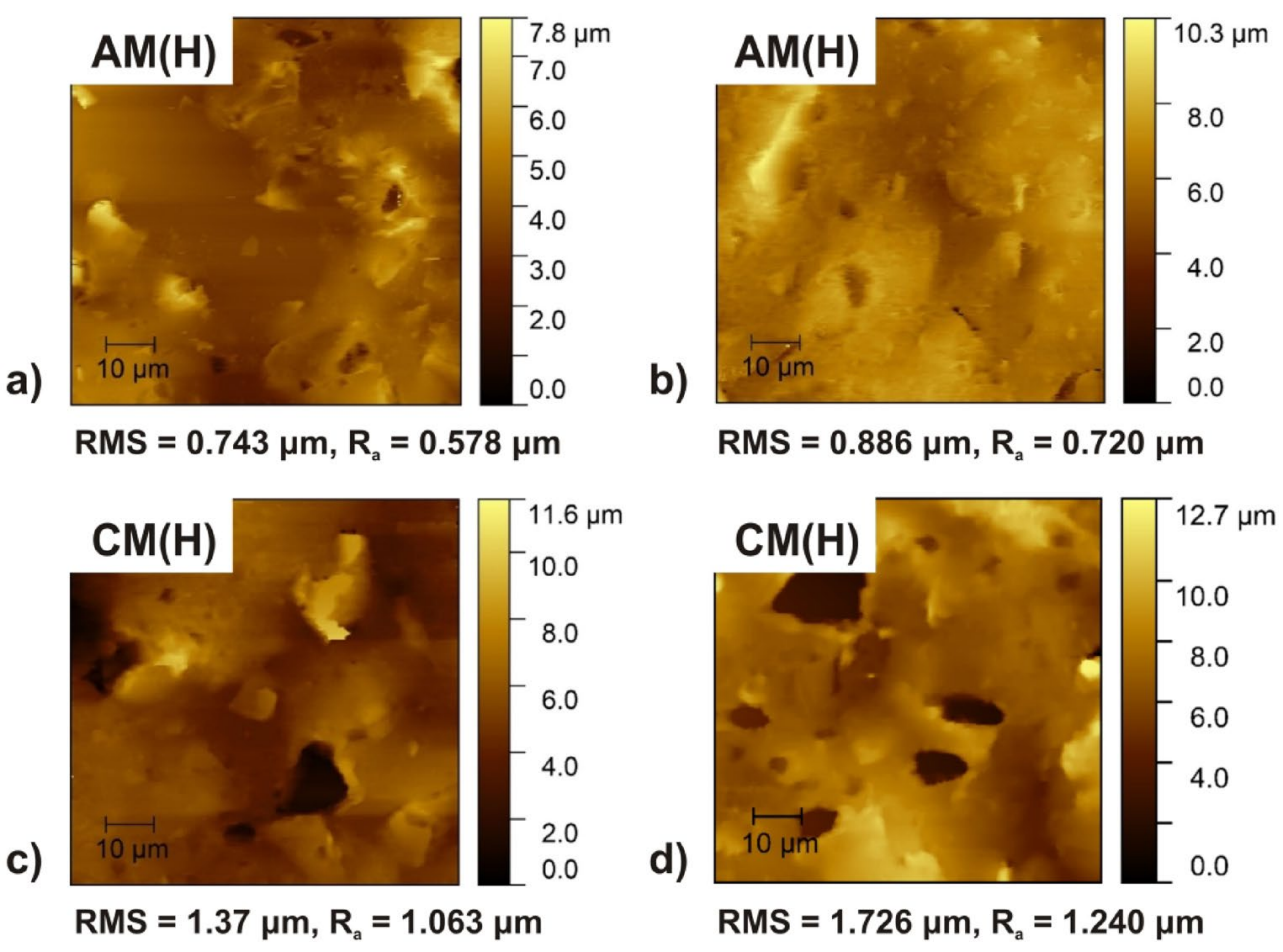
The AFM images of the tested AM(H) and CM(H) membranes: (**a**), (**c**) before and (**b**), (**d**) after ED.

In Fig. 9a, the surface morphology of the pristine IEMs is presented. As shown in Fig. 9a, the ion-exchanger particles were heterogeneously embedded in an inert membrane matrix. Thus, the presented membranes’ morphology proved their heterogeneity. Also AFM analysis of the pristine IEMs proved their heterogeneity (Fig. 10a, c). The AFM images of the pristine IEMs exhibited a developed heterogenous structure with micrometric roughness. The heterogeneously ion-exchanger grains and pores distributed in the membrane matrix are shown in Fig. 10a, c.

Heterogeneous IEMs, because of the presence of an inert polymer matrix, are characterized by a high mechanical strength and chemical stability. Moreover, if properly designed the IEMs’ inhomogeneity could improve the transport of ions across membranes. Thus, the heterogeneous membranes could be of interest for the successful treatment of corrosive wastewater, as well as ILs aqueous solutions. As the ion-exchange manufacturer (MEGA Inc., Czech Republic) assured us, the heterogeneous ion-exchange Ralex membranes are typically characterized by high mechanical and chemical stability as well as a long life cycle. In the data sheets of the tested AM(H) and CM(H) it is stated that they are resistant toward aggressive chemicals and fouling materials, as well as toward some industrial membrane poisons. Moreover, the tested membranes had long-term pH stability over a pH range of 0 to 14. Additionally, Ralex AM(H) and CM(H) membranes can be regenerated by alkaline and acidic chemicals. In the supplementary data file, Table S1, Ralex AM(H) and CM(H) membranes’ properties were added. Heterogeneous membranes are also cheaper than homogeneous membranes^48^. Therefore, the heterogeneous Ralex AM(H)-CM(H) IEMs were selected for the investigations.

During ED, fouling of IEMs can occur. Therefore, to identify the fouling formed on the membranes surfaces, SEM analysis of the IEMs was conducted before and after ED. In Fig. 9b, the surface morphology of the tested IEMs after five cycles of ED experiments are presented. It was noted that the surface morphology of the tested membranes after ED was not hardly changed compared with the morphology of pristine membranes (Fig. 9a). SEM images of the anion- and cation-exchange membranes after ED showed no mechanical cracks or damage. One commonly used parameter, which indicates changes in membrane surface structures, degradation, or changes in chemical composition, is the contact angle^49^. Figures 9c–d present the contact angle and wettability of the examined AM(H)-CM(H) IEMs before and after ED.

It was found that the wettability of the AM(H)-CM(H) membranes before and after the five cycles of experiments did not differ significantly. The contact angles of the pristine cation- and anion-exchange membranes were equal to 107.9° and 98.2°, respectively, while the contact angles of the cation- and anion-exchange membranes after ED were equal to 102.2° and 94.2°, respectively. Therefore, it was noted that after the experiment, the IEMs surface became more hydrophilic.

Also, the results of AFM analysis are in agreement with wettability test. The *R*_*a*_ of the pristine cation- and anion-exchange membranes were equal to 1.063 µm and 0.578 µm, respectively, while the *R*_*a*_ of the cation- and anion-exchange membranes after ED were equal to 1.240 µm and 0.720 µm, respectively (Fig. 10b, d). Thus, it was found that after ED the IEMs surface became slightly rough and in consequence more hydrophilic than the pristine samples due to the swelling of membranes and stretching of ion-exchanger grains and pores as a results of ED.

Additionally, the IEMs surfaces were examined by FTIR–ATR. In Fig. S1 the FTIR–ATR spectra of the tested CM(H) and AM(H) membranes before and after ED are presented. It was proven that no chemical change in the membrane surface was observed. The FTIR–ATR spectra of pristine membrane samples as well as membranes after the [Bmim]HSO_4_ recovery were similar and featured similar characteristic peaks (Fig. S1). The characteristic peaks at 2920 cm^−1^ and 2849 cm^−1^ were attributed to the stretching vibration of the C-H bond, corresponding to polyethylene (low density), which was used as a membranes inert binder. In the case of anion-exchange membrane after ED the band centered at 1073 cm^−1^ and the small shoulder at 998 cm^−1^ were assigned to symmetric stretching vibration of sulfate group^50^, which can be explained by sulfate anions transport across this membrane.

On the basis of our results, it was concluded that after five cycles of ED experiments, the tested heterogeneous membranes showed good durability and the ability to withstand acidic IL wastewater treatment. However, during prolonged membrane exposure to the IL, membranes fouling and lower efficiency and selectivity of the ED process can occur. Therefore, in the case of membrane fouling, a membranes cleaning strategy should be applied. Zhang et al.^51^ stated that their membrane cleaning process allows the membrane life-time to be enhanced. They described membrane cleaning consisting of stages involving cleaning with deionized water, then with 0.1M HCl, next with deionized water, then with 0.1M NaCl, and finally with deionized water, with a cleaning time of 30 min for each stage to allow for effective remove of membrane fouling^51,52^. Thus, to prolong the membranes’ performance, this cleaning method can be performed after each ED.

### Chemical stability of [Bmim]HSO_4_ in ED process with heterogeneous IEMs

As mentioned, the heterogeneous IEMs could be of interest for the successful treatment of corrosive wastewater, as well as ILs aqueous solutions. Moreover, heterogeneous membranes are cheaper than homogeneous membranes and have a high mechanical strength and chemical stability^48^. Shel’deshov et al.^53^ proposed that heterogeneous IEMs could be described as two-phase systems containing microporous gel regions, in which intergel spaces filled with an electrically neutral solution were embedded^53^. According to this model, the ionic moieties in the active layer of heterogeneous membrane are evenly distributed, but only in the gel regions, including polymer chains with fixed ion-exchange sites, whose charge is compensated for by mobile counterions^54^. Furthermore, it is assumed that^48,53,54^ these counterions can react with ILs during ED process by the salt metathesis reaction (i.e., double displacement reaction) between a solid phase and an IL. Therefore, ILs must be highly soluble in the process solutions^54^, so that IL ions cannot accumulate in the membrane material. In the present study, the [Bmim]HSO_4_ was soluble in all the process solutions during the experiments. Also, it was proven that the membrane surface morphology was not significantly changed in comparison to the pristine membranes (Figs. 9a–b). There was no deposition of solids and by-products on the membranes surface after ED.

**Fig. 11:**
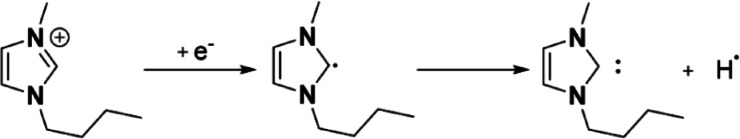
Reduction of [Bmim]^+^ through radical and carbene formation^55^.

Apart from the double displacement reaction in the membrane between IL ions and the ionic moieties in the active layer of the heterogeneous membrane, the chemical degradation of the imidazolium cation can be influenced by the presence of an acidic proton at the C2 position of the imidazole ring, which can undergo exchange even in neutral media (Fig. 11)^55^.

Moreover, a strongly basic anion as a counterion for [Bmim]^+^ may also increase the occurrence of such a degradation reaction. In the presence of a base, deprotonation of [Bmim]^+^ should occur even faster, giving a rich mixture of by-products. However, in this investigation, the pH of the electrolyte (0.1 M H_2_SO_4_) and [Bmim]HSO_4_ solutions in the ED chambers was acidic. Thus, the degradation mechanism of [Bmim]^+^ shown in Fig. 11 is less probable in the diluate and concentrate solutions. However, it is important to determine the chemical stability of [Bmim]HSO_4_ in the concentrate after ED.

**Fig. 12:**
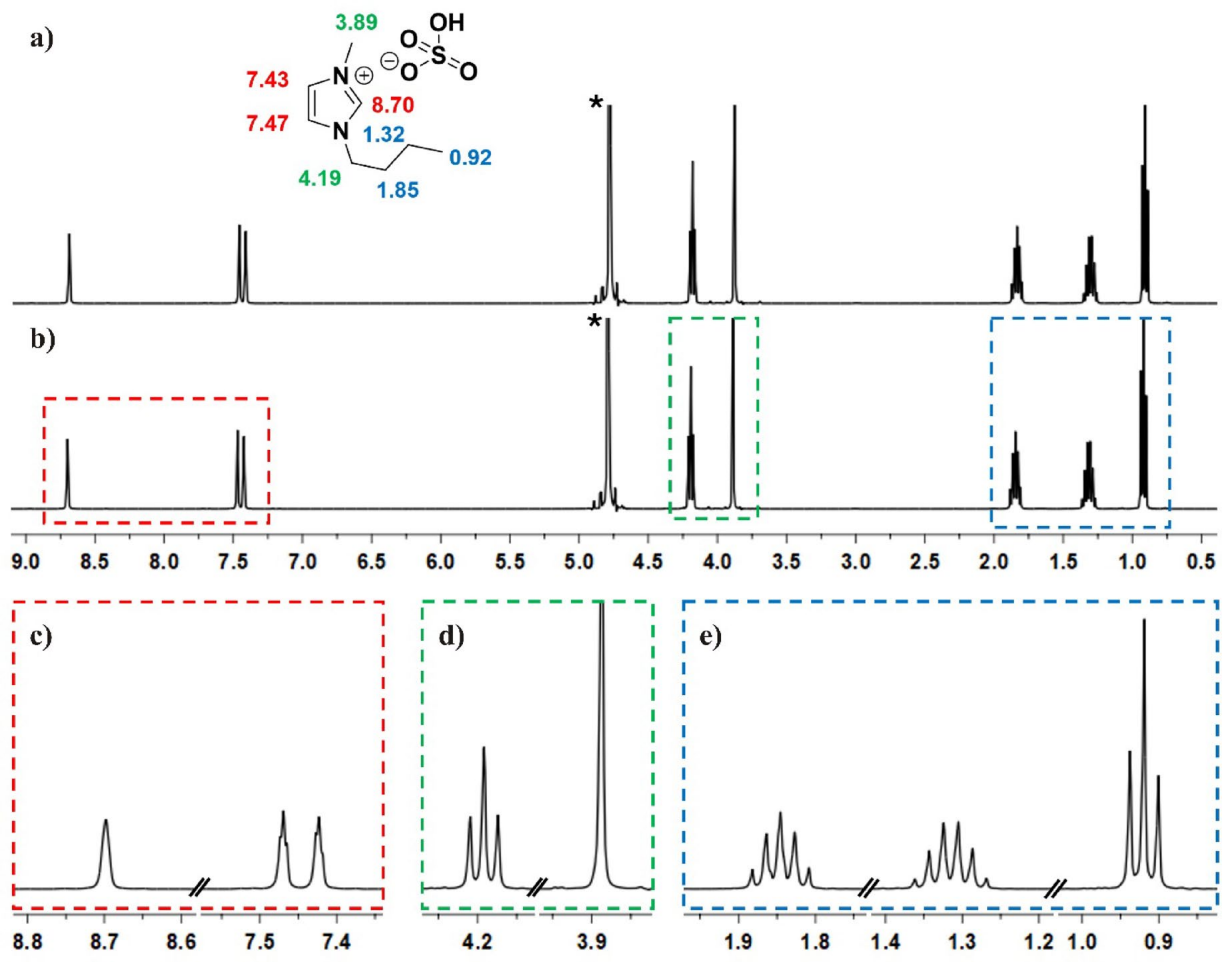
^1^H NMR spectra of [Bmim]^+^ process solution in D_2_O, pH 2.25; (**a**) blank—fresh solution of [Bmim]HSO_4_, (**b**) the concentrate solution after the ED process, (**c**) N–C*H* signals of imidazolium ring, (**d**) C*H* signals of imidazolium cation, (**e**) saturated alkane signals, *solvent signal.

Therefore, in this study, the chemical stability of the recovered [Bmim]^+^ was investigated to detect potential degradation products (Fig. 12). [Bmim]HSO_4_ in the model concentrate solution (Fig. 12a) and in the concentrate solution after the ED process (Fig. 11b) were determined using ^1^H NMR. In this case, D_2_O was directly added to sample(s) at various volumes to obtain 0.100 mmol/L of [Bmim]HSO_4_. The ^1^H NMR spectrum of the concentrate solution after ED was divided into three specific areas, i.e., 7.3–8.8 ppm: -C*H* signals of imidazolium ring (Fig. 12c), 3.4–4.3 ppm: N–C*H* signals of imidazolium cation (Fig. 12d), and 0.8–2 ppm: aliphatic chain, saturated alkane signals (Fig. 12e). NMR analysis confirmed the absence of degradable by-products of [Bmim]^+^ and its derivatives in the concentrate solution after ED (Fig. 12). In the range of the imidazole ring signals, no reduced intensity in the individual signal areas (Fig. 12c–e) and no signals from degradation products were observed. It is clear from Fig. 12 that the recovered [Bmim]HSO_4_ had the same structure to that of the fresh solution of [Bmim]HSO_4_. Therefore, it could be concluded that the recovered [Bmim]HSO_4_ was stable and could be reused.

### Future perspectives

Due to the wide applications of [Bmim]HSO_4_ in a variety of industrial fields, such as catalysis, biomass processing and electrochemistry, its high price, as well as toxicity, it is very important to develop this IL recovery and recycling methods^56,57^. Traditional methods of ILs recovery include vacuum distillation, extraction and adsorption, with vacuum distillation been the most common, as it allows for the removal of low boiling impurities and water^58–60^. However, this technique suffers from some serious disadvantages such as the possibility of decomposition of protic ILs containing sulfate anions at high temperatures, high energy consumption, and large operating costs^58^. Therefore vacuum distillation is preferably applied for the recovery of ILs from wastes with high ILs concentrations. More thermally sensitive ILs can be recovered by extraction or adsorption^59,60^. Extraction relies on the difference in solubility of the separated compounds in two immiscible liquid phases, and due to the strong solubility of ILs in water, extraction is not a preferred method for separation of these compounds from aqueous solutions. ILs can be successfully recovered by adsorption, however, its use is limited to solutions with low ILs concentrations, mainly due to the limited sorption capacity of the available solvents. Despite the above, sorbents such as soils, activated carbons, macroporous resins, montmorillonite, bentonite, clays, ion-exchange resins, or biosorbents can be successfully applied for recovery of ILs from wastes^59,60^. Adsorption, is known to be more effective in separation of ILs, however, the desorption of the ILs from the above-mentioned sorbents can be still challenging^58–60^. Electrodialysis constitute the more selective, energy efficient, and environmentally sustainable alternative for [Bmim]HSO_4_ recovery from aqueous solutions compared to above-discussed techniques^57,59^. In comparison to vacuum distillation, the great advantage of ED lies in the fact that it does not require heating. Unlike solvent extraction and sorption ED does not require the use of additional organic solvents or leaching solutions, thus it limits environmental and safety hazards related to those compounds^59,60^. Moreover, ED as a modular membrane separation technique is known to be easily scalable, i.e. by increasing the number of membrane pairs per stack or by combining separate ED modules in-parallel (or less commonly in-series) with no considerable loss in performance and efficiency. However, along with ED, as with any other membrane method, comes a serious question of membrane stability and chemical resistance^28,35^. With that in mind we applied heterogeneous IEMs, which are known to be successfully applied for the successful treatment of harsh, corrosive and strongly acidic solutions. Moreover, the heterogeneous membranes applied herein are characterized by excellent mechanical and chemical stability as well as a long life-cycle. The comparison of advantages and disadvantages of ED as an ILs recovery method with other was summarized in Table 3. In future perspectives to maximize these advantages the research on the ED in combination with other processes, such as adsorption, extraction and vacuum distillation might also be considered.

**Table 3:** Comparison of electrodialysis with other ILs recovery methods^58–60^.

Methods	Advantages	Disadvantages
Electrodialysis	High selectivity Allow for simultaneous ILs concentration and recovery Osmotic pressure is not a limited factor Relatively low energy consumption Modularity and scalability Potential to reuse of the secondary stream Possibility of application the heterogeneous ion-exchange membranes for harsh feed solution	Cost of ion-exchange membranes Membrane fouling can occur
Vacuum distillation	Short processing cycle Simple operating method No require the use of toxic reagents Suitable for recovery high concentration ILs	High operating costs Energy-intensive Thermal decomposition of ILs can occur
Extraction	Energy efficient Simple operating method Simple equipment Suitable for thermal sensitive ILs	Requires solvent selection, which allows for efficient and selective ILs recovery without their chemical decomposition Cost of solvent Solvent recycling is necessary Disposal of spent extractant May not be environmental friendly
Adsorption	Suitable for very diluted solution Simple operating method	Requires adsorbent selection, recovery of ILs depends on the physicochemical properties of adsorbents (pore size, functional group) Slow ILs diffusion Limited lifespan of adsorbent Regeneration of adsorbent Require desorption stage with desorption solvent selection Desorption may be difficult and incomplete

The lab-scale experiments discussed in this work should be helpful for better understanding and approximating applicability of the ED process for ILs separation, as well as give some insight and perspectives of the proposed method. However, testing the effectiveness of electrodialytic recovery of [Bmim]HSO_4_ in a real-world conditions seems to be important for the technology scale-up. Therefore, the further research line, that we will be developed in future work, is to test the removal of this IL from real wastewaters at a pilot scale.

As shown in this study, application of ED with heterogeneous ion-exchange membranes allowed for the simultaneous recovery and concentration of [Bmim]HSO_4_ from wastewaters, which might significantly alleviate its potential harm to the environment and wastewater storage or disposal. Moreover, the possibility of reusing the concentrate obtained in the ED process brings both ecological and economic benefits.

In future we plan to assess the whole environmental impact of the examined [Bmim]HSO_4_ recovery method, including energy consumption, water and chemical usage, waste generation on the industrial scale, along with the life cycle assessment (LCA) analysis.

## Conclusions

Electrodialysis is an electromembrane process, which allows ionic species to be transported through IEMs. In this study, [Bmim]HSO_4_ recovery from wastewater was successfully achieved by ED with heterogeneous IEMs. The effects of the IL concentration in the diluate solution, the applied voltage, and the linear flow velocity on the ED performance and [Bmim]HSO_4_ recovery were discussed.

It was proven that the initial concentration of [Bmim]HSO_4_ in the feed solution influenced the effectiveness of the electrodialytic IL recovery. It was found that the [Bmim]HSO_4_ recovery ratio, the [Bmim]^+^ molar flux, the *CE*, and the *EC* increased with the increasing initial concentration of [Bmim]HSO_4_ in the feed solution from 0.01 to 0.2 mol/L. When the [Bmim]HSO_4_ concentration in the feed was 0.2 mol/L, 98.8% of [Bmim]HSO_4_ was recovered with a *CE* of 67.4% and an *EC* of 28 kWh/m^3^. The ED process allowed not only for [Bmim]HSO_4_ recovery, but also for the concentration of the [Bmim]HSO_4_. The [Bmim]HSO_4_ content in the concentrate solution after ED of the 0.2 M feed solution increased 2.3 times in comparison to the [Bmim]HSO_4_ concentration in the feed. Moreover, the analysis of the influence of the applied voltage in this study showed that the [Bmim]HSO_4_ recovery, its degree of concertation, the *CE*, and the *EC* increased as the applied voltage increased. It was also noted that the selection of the appropriate diluate flow rate was critical. A higher linear flow velocity can reduce the concentration polarization and increase the ED efficiency; however, it can also negatively affect the removal efficiency by reducing the residence time of ions in the compartment, and it leads to a decrease in the total ions transport across IEMs. Based on the obtained results, it was proven that the highest ED efficiency for the recovery and concentration of [Bmim]HSO_4_ was obtained at an applied potential of 4 V, a 2 cm/s linear flow velocity, and 0.2 M IL in the feed solution. Furthermore, it was shown that the structure of the recovered [Bmim]HSO_4_ solution did not differ in comparison to the fresh [Bmim]HSO_4_ solution.

To sum up, it can be concluded that ED can be considered as an effective method for recovering [Bmim]HSO_4_ from aqueous solution. Thus, the presented results provide insights for developing the electrodialytic recovery method of [Bmim]HSO_4_. Accordingly, the presented results could allow the application range of ED to be broadened to become a recovery method for acidic ILs in the future.

## Electronic supplementary material

Below is the link to the electronic supplementary material.

Supplementary Material 1

## Data Availability

The data and material generated during and/or analyzed during the current study are available from the corresponding author upon reasonable request.

## Abbreviations

*AFM*: Atomic force microscopy
[Bmim]HSO_4_: 1-Butyl-3-methylimidazolium hydrogen sulfate
$CE_{IL}$: Electric current efficiency (%)
$C_{IL,0}^{{conc_{{}} }}$: The initial concentration of the [Bmim]HSO_4_ in the concentrate solution before ED (mol/L)
$C_{IL,t}^{conc}$: The final concentration of the [Bmim]HSO_4_ in the concentrate solution after ED (mol/L)
*EC*: Energy consumption (kWh/m^3^)
ED: Electrodialysis
*F*: Faraday constant 96,485 (C mol^−^^1^)
*FTIR*: Fourier transform infrared spectroscopy
*I*: Electric current (A)
*IEMs*: Ion-exchange membranes
*IL*: Ionic liquid
$M_{IL}$: The molar mass of [Bmim]HSO_4_ (g/mol)
$m_{IL,t}^{conc}$: The increase in the [Bmim]HSO_4_ mass in the concentrate solution after ED (g)
$m_{IL,0}^{dil}$: The initial mass of the [Bmim]HSO_4_ in the diluate solution before ED (g)
*n*: Number of membrane pairs
*NMR*: Nuclear magnetic resonance
R_a_: Roughness average (µm)
*R*_conc_: The [Bmim]HSO_4_ degree of concentration
$R_{IL}$: The [Bmim]HSO_4_ recovery ratio (%)
*RMS*: Root mean square roughness (µm)
SEM: Scanning electron microscopy
*t*: Time (min)
*U*: Applied voltage (V)
$V_{conc,t}^{{}}$: Volume of the concentrate solution after ED (L)
$V_{dil,0}^{{}}$: Initial diluate volume (m^3^)
*w*: Linear flow velocity (cm/s)
*z*: Charge number of [Bmim]^+^
