# Supplementary material for: Insights into 1-butyl-3-methylimidazolium hydrogen sulfate recovery from wastewater by electrodialysis with heterogeneous ion-exchange membranes
Source: Sci Rep. 2025 Jun 2;15:19299. doi: 10.1038/s41598-025-04108-y (PMC12130326; doi:10.1038/s41598-025-04108-y)
Supplement: Supplementary file 1 — Supplementary Material 1 [file 41598_2025_4108_MOESM1_ESM.pdf]

Insights into 1-butyl-3-methylimidazolium hydrogen sulfate recovery from wastewater by electrodialysis with heterogeneous ion-exchange membranes

Dorota Babilas-Krzyżowska[a*], Jitka Chromikova[b], Andrzej Milewski[a], Sandra Pluczyk-Małek[c,d], Piotr Dydo[a]

[a] Department of Inorganic, Analytical Chemistry and Electrochemistry, Faculty of Chemistry, Silesian University of Technology, B. Krzywoustego 6 Street, 44-100 Gliwice, Poland, *Corresponding author email: dorota.babilas@polsl.pl

[b] Department of Environmental Engineering, Faculty of Mining and Geology, VŠB – Technical University of Ostrava, 17. Listopadu 15 Street, Ostrava-Poruba, Czech Republic

[c] Department of Physical Chemistry and Technology of Polymers, Faculty of Chemistry, Silesian University of Technology, M. Strzody 9 Street, Gliwice 44-100, Poland

[d] Centre for Organic and Nanohybrid Electronics, Silesian University of Technology, S. Konarskiego 22B Street, Gliwice 44-100, Poland

Table S1. Characteristics of Ralex AM(H)-CM(H) (Mega Inc., Czech Republic) ion-exchange membranes[1–3].

**Membrane properties**	**AM(H)**	**CM(H)**
Morphology	heterogeneous	heterogeneous
Type	anion-exchange	cation-exchange
Fitting fabrics	polypropylene	polypropylene
Basic binder on base	polyethylene	polyethylene
Transport number (0.5/0.1M KCl)	> 0.95	> 0.95
Exchange capacity, meq/g	1.8	2.2
Long-term pH stability scale	0-14	0-14
Thickness of swelled membrane, [mm]	< 0.70	< 0.70
Thermal resistance under DC current	40°C	40°C
Resistance against aggressive chemicals and fouling materials	resistant	resistant
Possibility of frequent regeneration by acidic and alkaline chemicals	regeneration possible	regeneration possible
Regeneration, sanitation	up to 1 hour: 90°C, more than 1 hour: 65°C	up to 1 hour: 90°C, more than 1 hour: 65°C

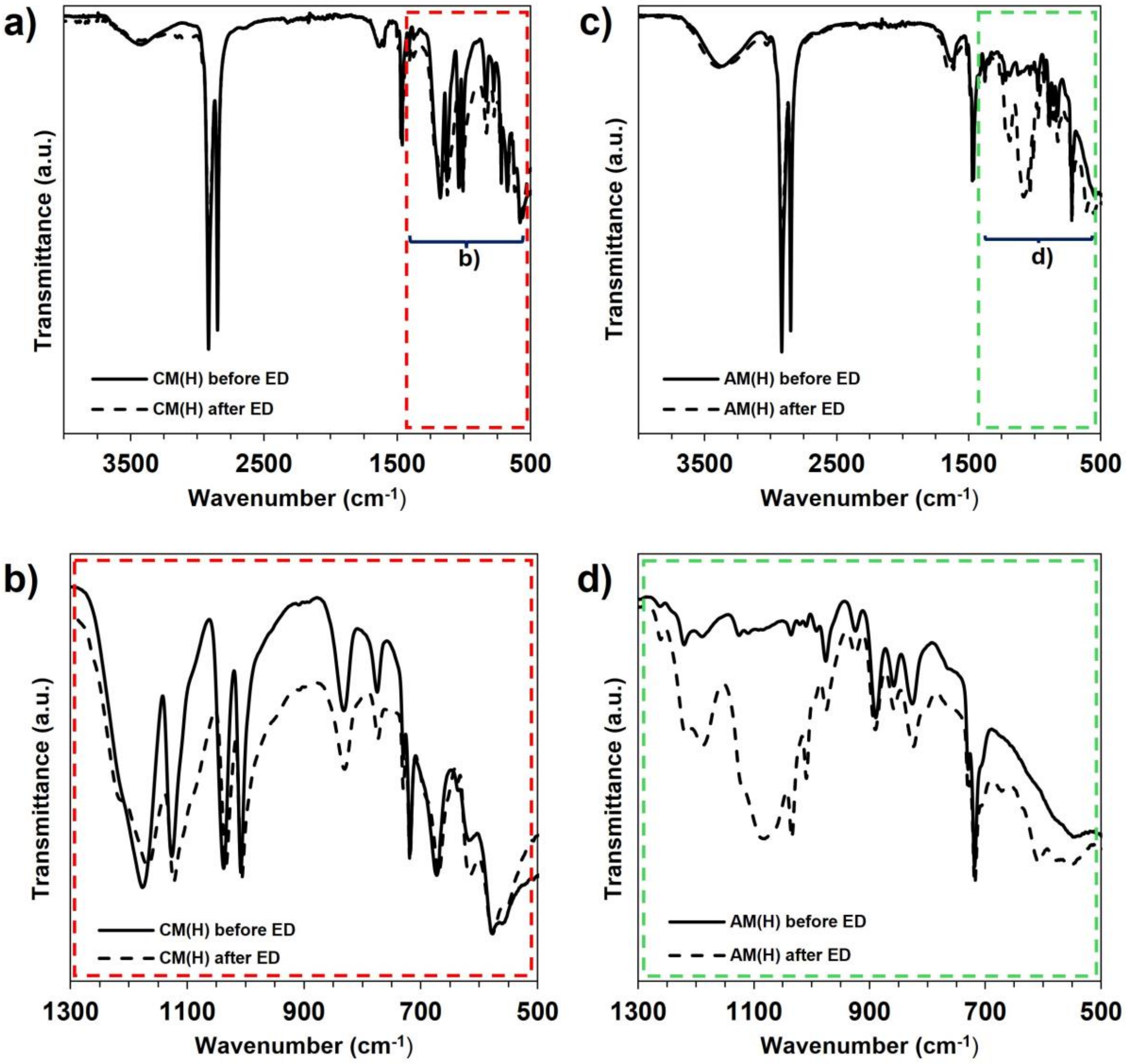

Figure S1. The FTIR–ATR spectra of the tested CM(H) and AM(H) membranes before and after ED: (**a**), (**c**) wavenumber from 4000 cm^{-1} to 500 cm^{-1}; (**b**), (**d**) wavenumber from 1300 cm^{-1} to 500 cm^{-1}.

References

1. Ralex membrane AMHPP product data sheet. https://www.mega.cz/files/datasheet/MEGA-RALEX-AMH-PP-en.pdf (2025).
2. Ralex membrane CMHPP product data sheet. https://www.mega.cz/files/datasheet/MEGA-RALEX-CMH-PP-en.pdf (2025).
3. Mohamed, A.Sh., El-Shamy, A., Emara, M. & Shahba, R.M.A. Application of electrodialysis technique in removal of some heavy metal ions from discharge wastewater in paper industry. *Al-Azhar Bull. Sci.* **32**, 1–9 (2021).
